# Primary lymphoma of mucosa associated lymphoid tissue (MALT lymphoma) in the urinary bladder mimicking recurrent urinary tract infection: a case report and literature review

**DOI:** 10.1186/s12894-024-01616-3

**Published:** 2024-10-12

**Authors:** Po-Sung Liang, Hung-Jen Shih, Sheng-Hsien Huang, Yi-Zhong Chen

**Affiliations:** 1 Division of Urology, Department of Surgery, Changhua Christian Hospital, Changhua City, Changhua County, Taiwan; 2 Department of Post-Baccalaureate Medicine, College of Medicine, National Chung Hsing University, Taichung, Taiwan; 3 Department of Urology, School of Medicine, College of Medicine, Taipei Medical University, Taipei, Taiwan

**Keywords:** Bladder lymphoma, Mucosa associated lymphoid tissue lymphoma, Case report

## Abstract

We report the case of a 79-year-old woman with primary lymphoma of mucosa-associated lymphoid tissue (MALT) in the urinary bladder. The patient, with urinary frequency, urgency and suprapubic pain had several emergency room visits due to recurrent urinary tract infection. Both sonogram and cystoscopy identified bladder tumors near the bladder neck. An abdominal contrast-enhanced computed tomography scan revealed a polypoid lesion on the anterior bladder wall without enlarged lymph nodes. Transurethral resection of the bladder tumor was conducted. The pathology report confirmed extranodal marginal zone MALT lymphoma. The clinical stage was IEA. Follow-up imaging reported residual bladder tumors, prompting adjuvant radiotherapy. The patient was treated successfully and was disease-free at the 9-month follow-up visit. Primary lymphoma is an uncommon pathological subtype. Its clinical and radiological differentiation from urothelial carcinoma (UC) can be challenging, but treatment strategies differ significantly. A definitive diagnosis relies on histopathology and immunohistochemistry. Typically, bladder lymphoma has a favorable prognosis, but further research is required to identify the optimal treatment.

## Introduction

Primary lymphoma in the urinary bladder is rare, and its definitive diagnosis relies on histopathology and immunohistochemistry. We present the case of a 79-year-old woman with primary lymphoma of mucosa-associated lymphoid tissue (MALT) in the urinary bladder, mimicking urinary tract infection (UTI). The collaborative and multifaceted approach described in this study aims to provide the most appropriate and effective treatment for her rare condition.

### Case presentation

**Fig. 1:**
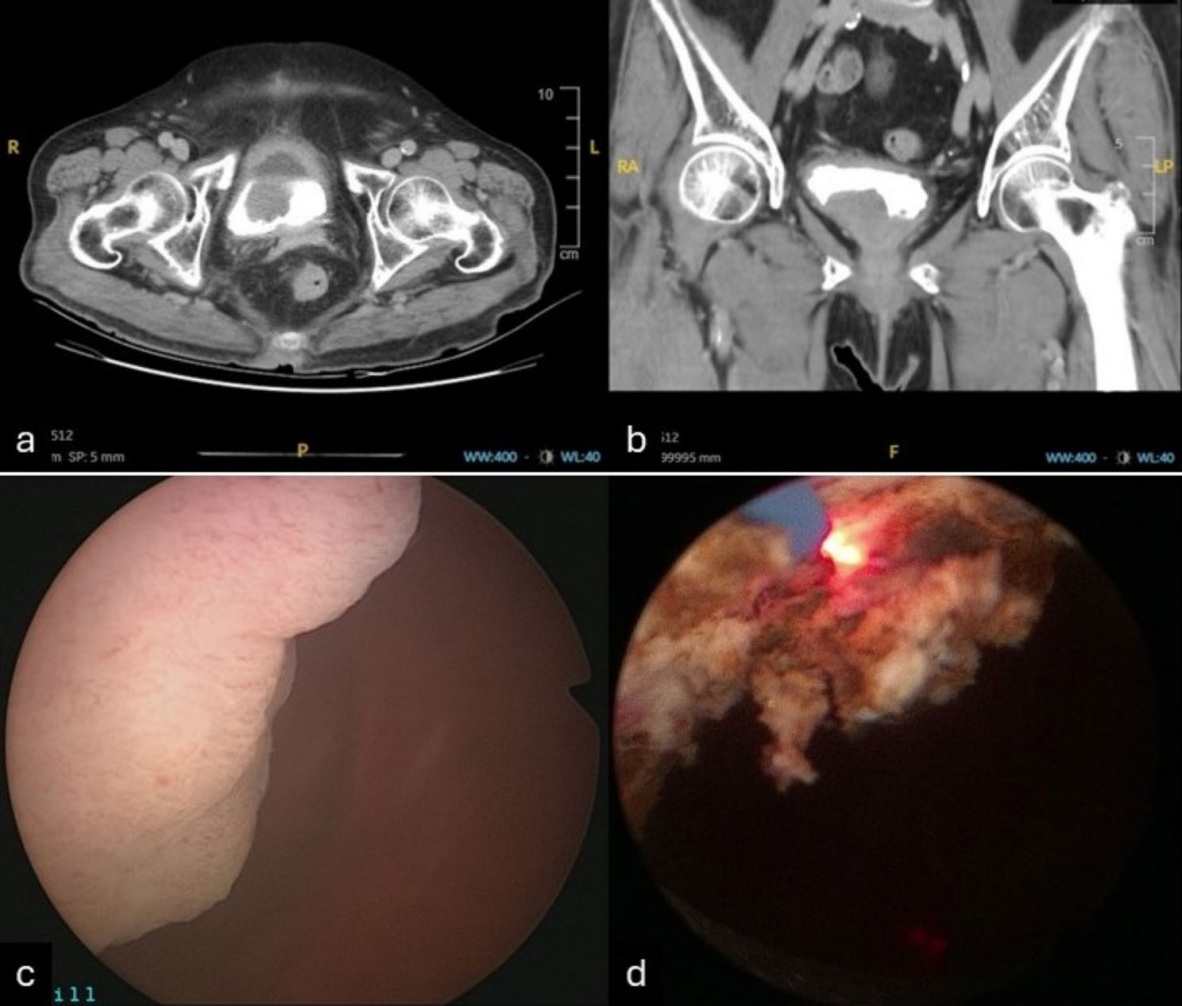
(**a**-**b**) The CT revealed a 3.9 cm mass on the anterior wall of the bladder. (**c**-**d**) During TURBT, the bladder tumors on the anterior wall near bladder neck were resected using a thulium laser under an Fr. 27 transurethral endoscope

**Fig. 2:**
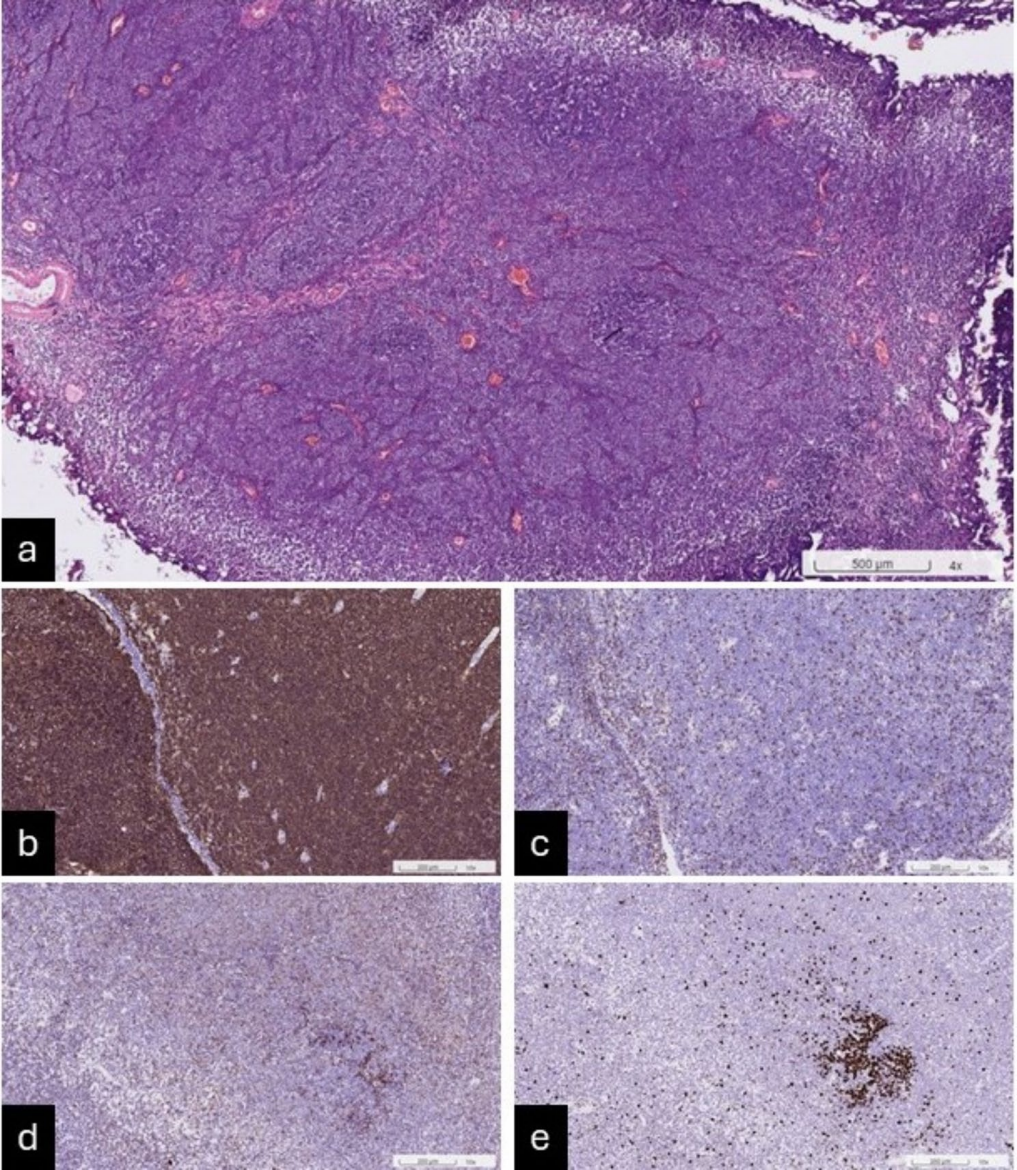
(**a**) Microscopically, the urinary bladder shows diffuse infiltrate of morphologically heterogeneous small B cells. The tumor cells have irregular angulated nuclear contours with inconspicuous nucleoli and pale cytoplasm. (**b**) Positive for CD20 (marker of low-grade lymphoma). (**c**) Negative for CD3 (marker of high-grade lymphoma). (**d**) Negative for BCL6. (**e**) Ki-67 proliferation index was low

A 79-year-old woman with a history of hypertension and diabetes mellitus presented to our urologic clinic with urinary frequency and urgency, as well as suprapubic pain. She had been experiencing the symptoms for 6 months and visited the emergency room multiple times over this period due to recurrent urinary tract infections (UTIs). No significant findings were observed during the physical examination. The initial urinalysis conducted at the time of the primary visit revealed a red blood cell count of 9 /µl and a white blood cell count over 500 /µl. The urine culture tests consistently revealed the presence of *Escherichia coli*. Upon her arrival at our outpatient department, she underwent a sonogram, reporting left renal cysts and a suspicious 3.2-cm bladder tumor. During cystoscopy, multiple bladder tumors were identified, predominantly in the anterior wall of the urinary bladder, near the neck. A biopsy was conducted, and the pathology results revealed atypical lymphoid infiltration. A high-contrast abdominal computed tomography (CT) scan revealed a 3.9-cm enhancement, identified as a polypoid lesion, on the anterior wall of the urinary bladder [Fig. 1a-b]. Lymph nodes were not enlarged. A transurethral resection of the bladder tumor (TURBT) was conducted, where the bladder tumors were resected using a thulium laser under an Fr. 27 transurethral endoscope [Fig. 1c-d]. On gross examination, the specimens were brownish and elastic tissue fragments. Microscopically, the urinary bladder revealed a widespread presence of various types of small B cells, such as marginal zone cells resembling centrocytes, monocytoid cells, small lymphocytes, immunoblasts, and centroblast-like cells. Additionally, a few nonneoplastic germinal centers are also observed. The tumor cells display uneven and angled nuclear shapes with indistinct nucleoli and pale cytoplasm [Fig. 2a]. The pathology report confirmed the diagnosis of extranodal marginal zone lymphoma of MALT. Immunohistochemical staining was positive for CD20 and BCL2 but negative for CD3, BCL6, CD10, MNDA, CD5, CD23, and cyclin D1 [Fig. 2b-d]. The patient’s ki-67 proliferation index was low [Fig. 2e]. We referred her to the Hemato-oncology Department for a bone marrow biopsy. The resulting pathology report disclosed no evidence of malignancy. The whole body positron emission tomography was prepared and conducted, revealing no definite abnormal fluorodeoxyglucose metabolic lesions of the head, neck, chest, abdomen, pelvis, or bones. The comprehensive staging work-up indicated that the patient was at clinical stage IEA of her disease according to the Lugano classification for lymphoma staging. However, follow-up imaging revealed some residual bladder tumors. To provide local control, adjuvant radiotherapy was initiated. She underwent 20 sessions of radiation therapy, receiving a total dose of 2400 cGy specifically targeting the urinary bladder. Chemotherapy was not administered. Her recovery after irradiation was smooth, with only a slight discomfort during urination. The urinalysis conducted after treatment showed a red blood cell count of 2 /µl and a white blood cell count of 5 /µl. No bacterial growth was detected in the post-therapy urine culture. She attains a complete response on imaging and cystoscopy during 9 months of follow-up. At present, she is attending follow-up appointments in our outpatient department.

## Discussion

Primary lymphoma of the urinary bladder is a rare occurrence, accounting for approximately 1.0% and 0.2% of all bladder tumors and extranodal non-Hodgkin’s lymphomas [1]. It typically manifests with intermittent gross hematuria, urinary frequency and urgency, dysuria, nocturia, and suprapubic and abdominal pains [2]. Common risk factors include chronic inflammation, UTIs, and autoimmune diseases. Approximately 20% of cases have a history of chronic bladder inflammation [3], leading to the hypothesis that chronic cystitis is potentially associated with an increase in extranodal lymphoid tissue, ultimately progressing to lymphoma [2]. Regarding the association between *Helicobacter pylori* infection and gastric MALT lymphoma, the regression of bladder MALT lymphoma postantibiotic treatment of *Escherichia coli*-related UTIs has been documented by Oscier et al. [4]. This lymphoma type is frequently observed dome or lateral walls of the bladder. Typically, it does not invade the ureteral orifice or spread throughout the bladder, making hydronephrosis an uncommon clinical presentation [5].

Distinguishing the clinical symptoms and imaging findings of primary lymphoma of the urinary bladder from urothelial carcinoma (UC) can be challenging. Therefore, diagnosing these tumors often relies on histopathology and immunohistochemistry. Strong positive immunohistochemistry staining for the markers LCA and CD20 indicates B-cell-derived lymphoma, while negative staining for cytokeratin (CK), CK7, and CD45RO excludes various carcinomas. Lymphomas of the bladder are categorized into low- and high-grades. Low-grade lymphomas produce positive stains for the cell markers CD20, CD43, and CD21, while high-grade lymphomas for CD20, CD3, and CD30 [6]. The most common low-grade type is B-cell-derived non-Hodgkin’s lymphomas of MALT, which includes Burkitt’s and plasmacytoid lymphomas [1]; these tend to have long disease courses and insidious onset but favorable prognosis [2]. Furthermore, MALT lymphomas produce positive stains for CD19 and FMC7, and negative for CD5, CD10, and CD11c [5]. The most common high-grade type is diffuse large B-cell lymphoma (DLBCL) [1]; further imaging studies such as chest and abdominal CT scans, bone marrow aspiration evaluations, and bone biopsies are recommended to rule out systemic lymphoma.

Table 1 presents the epidemiology, clinical features, and therapies of the cases of primary lymphoma of the urinary bladder as studied from the literature. Previously documented cases exhibit a varied array of clinical features, therapies, and prognosis. The majority of patients were female, mostly over the age of 50, with hematuria being the most prevalent presenting symptom, followed by dysuria, urinary frequency, and recurrent UTIs. Treatments included chemotherapy, radiotherapy, and TURBT, with numerous cases demonstrating a positive outcome. In numerous instances, diverse chemotherapy protocols were utilized, and radiation therapy was frequently administered. TURBT served as a therapeutic alternative in certain instances, especially for localized illness. In one case, antibiotic therapy resulted in complete remission. The follow-up durations varied from several months to several years, with numerous patients being alive and disease-free at the time of assessment, suggesting a predominantly positive prognosis when treated adequately. Nonetheless, certain instances, especially those concerning DLBCL variations or associated problems, led to recurrence or mortality, highlighting the diversity in outcomes contingent upon lymphoma subtype and treatment approach.

**Table 1:** Epidemiology, clinical features, therapies and outcomes of the cases of primary lymphoma of the urinary bladder

Paper	Age	Sex	Histology	Presenting symptoms	Treatment	Outcome
Leite et al.	89	Female	Diffuse Large B-cell Lymphoma	Urinary obstruction	Chemotherapy (CHOP)*6	Recurrence and death after 1 year
Xu et al.	77	Female	MALT Lymphoma	Dysuria, urinary frequency and urgency	TURBT	Alive and healthy at 15-month follow-up
Aceñero et al.	73	Female	MALT Lymphoma	Pain over left lumbar area, dysuria	Chemotherapy (unknown regimen)	Death 8 months later due to unrelated cause. Autopsy showed no recurrence in bladder, but lymphoma of the thyroid.
	50	Female	MALT Lymphoma	Fever	Chemotherapy (unknown regimen)	Alive and free of disease after 5 years
	75	Female	MALT Lymphoma	Hematuria, dysuria	Chemotherapy (unknown regimen)	Alive and free of disease after 9 months
Oscier et al.	78	Female	MALT Lymphoma	Recurrent UTI	Antibiotic therapy for UTI	Complete remission
Vempati et al.	65	Female	MALT Lymphoma	Hematuria	Radiation (30 Gy in 20 fractions)	No evidence of disease at 3-month follow-up
Bates et al.	66	Female	MALT Lymphoma	Bladder mass	?	Alive and free of disease after 1 year
	79	Female	MALT Lymphoma	Hematuria	?	No follow-up
	59	Female	MALT Lymphoma	?	?	Alive with disease after 3 years
	84	Female	Diffuse Large B-cell Lymphoma	Hematuria	?	Alive with disease after 16 years
	67	Male	Diffuse Large B-cell Lymphoma	Hematuria	Radiation + chemotherapy (unknown regimen)	Alive and free of disease after 3 years
	80	Female	Diffuse Large B-cell Lymphoma	Hematuria	Radiation	Alive and free of disease after 1 year
Hughes et al.	82	Female	MALT Lymphoma	Hematuria	Radiation + chemotherapy (ChlVP)	Death
	81	Female	MALT Lymphoma	Hematuria	TURBT	Alive and free of disease after 1 year
	28	Male	MALT Lymphoma	Hematuria	Chemotherapy (ChlVP)*6	Alive and free of disease after 10 years
	76	Female	MALT Lymphoma	Hematuria	Radiation	Alive and free of disease after 2 years
	77	Male	MALT Lymphoma	Hematuria	Chemotherapy (ChlD)*6	Alive and free of disease after 4 years
	66	Female	MALT Lymphoma	Recurrent UTI	Radiation	Death
	31	Male	Diffuse Large B-cell Lymphoma	Recurrent UTI	Doxycycline	Alive and free of disease after 8 years
	71	Female	Diffuse Large B-cell Lymphoma	Hematuria	Chemotherapy (CHOP)*6	Alive and free of disease after 2 years
	70	Female	Diffuse Large B-cell Lymphoma	Hematuria	Suregery + chemotherapy (CHOP)*6	Alive and free of disease after 4 years
	75	Female	Diffuse Large B-cell Lymphoma	Hematuria	Chemotherapy (IChlP)	Death
Aando et al.	77	Female	MALT Lymphoma	Urinary retention	TURBT	Alive and free of disease after 3 years
Yener et al.	56	Male	MALT Lymphoma	Dysuria, periumbilical pain	Rituximab*6	Alive and free of disease after 5 years
Hatano et al.	84	Female	MALT Lymphoma	Hematuria, UTI	Radiation	Alive and free of disease after 1 year
Evans et al.	64	Female	Diffuse Large B-cell Lymphoma	Recurrent UTI	Chemotherapy (R-CHOP)	Treatment on going
Horasani et al.	65	Female	Diffuse Large B-cell Lymphoma	Hematuria, dysuria, urinary frequency	Radiation + chemotherapy (CHOP)*4	Alive and free of disease after 1 year
Simpson et al.	48	Male	Diffuse Large B-cell Lymphoma	Recurrent UTI	Chemotherapy (R-CHOP)*6	Alive and free of disease after 6 months

MALT: mucosa associated lymphoid tissue; UTI: urinary tract infection; CHOP: Cyclophosphamide, Daunorubicin, Vincristine, Prednisolone; Chl: Chlorambucil; V: Vincristine; P: Prednisolone; D: Dexamethasone; I: Idarubicin; R: Rituximab

Based on the current National Comprehensive Cancer Network (NCCN) guideline, the recommended first-line treatment for extranodal marginal zone lymphoma occurring outside of the stomach and skin is involved-site radiation therapy. TURBT may be used as a diagnostic and therapeutic procedure. Reportedly, the extent of surgical resection, whether it is total cystectomy, partial cystectomy, complete TURBT, or incomplete TURBT, does not significantly affect the overall prognosis. Moreover, the choice of surgical intervention may not be a major determinant of clinical outcomes. Rather, the primary goal of surgical intervention is to alleviate lower urinary tract symptoms [5]. In cases of local recurrence, it is advisable to contemplate the use of systemic chemotherapy, while radiation therapy can be a palliative approach to achieve local control. Patients experiencing systemic recurrence are treated similarly to those with nodal lymphomas, with systemic chemotherapy being the subsequent therapeutic choice. While there are no established guidelines on the most effective regimen for lymphoma of the bladder, CHOP (cyclophosphamide, hydroxydaunorubicin, oncovin and prednisolone) is commonly employed for four cycles. Incorporating rituximab into the treatment regimen is recommended, particularly for individuals who exhibit resistance to chemotherapy and have a BCL-2 gene translocation [7]. The mitochondrial pathway contributes to the development of non-Hodgkin’s lymphoma, and rituximab has been demonstrated to alleviate the chemotherapy resistance observed in individuals with BCL-2 overexpression. This is achieved by stimulating cell apoptosis through the BCL-2 regulated mitochondrial pathway [8]. Clinical observations of MALT lymphomas indicate that they typically manifest as indolent diseases with slow dissemination rates. The 10-year survival rate is approximately 90% [9]. To date, there are no formal guidelines on disease surveillance. Vempati et al. suggest annual cystoscopic monitoring for the first 2– 3 years with urine specimens obtained at each visit for cytology [10].

## Conclusion

Among bladder tumors, primary lymphoma is a relatively rare and less malignant pathological subtype. Urologists should keep this less common diagnosis in mind before surgery. It is difficult to distinguish clinically and radiologically from UC, but treatment strategies differ significantly. An accurate diagnosis relies on histopathology and immunohistochemistry. More study is needed to determine the optimal treatment and prognosis.

## Data Availability

All data and figures produced or examined during this investigation are contained within this published paper and its additional material files.

## References

[1] Leite KR, Bruschini H, Camara-Lopes LH. Primary lymphoma of the bladder. Int Braz J Urol. 2004;30:37–9. 10.1590/s1677-55382004000100009. 15707515 10.1590/s1677-55382004000100009

[2] Xu H, Chen Z, Shen B, Wei Z. Primary bladder mucosa-associated lymphoid tissue lymphoma: a case report and literature review. Medicine. 2020. 10.1097/MD.0000000000020825. 99.28. 32664075 10.1097/MD.0000000000020825 PMC7360252

[3] Aceñero MF, Rodilla CM, García-Asenjo JL, Menchero SC, Esponera JS. Primary malignant lymphoma of the bladder. Report of three cases. Pathol Res Pract. 1996;192:160–3. 10.1016/S0344-0338(96)80211-1. 8692717 10.1016/S0344-0338(96)80211-1

[4] Oscier D, Bramble J, Hodges E, et al. Regression of mucosa-associated lymphoid tissue lymphoma of the bladder after antibiotic therapy. J Clin Oncol. 2002;20:882. 10.1200/JCO.2002.20.3.882. 11821481 10.1200/JCO.2002.20.3.882

[5] Venyo AK. Lymphoma of the urinary bladder. Adv Urol. 2014;2014:327917. 10.1155/2014/327917. 24511310 10.1155/2014/327917 PMC3912819

[6] Bates AW, Norton AJ, Baithun SI. Malignant lymphoma of the urinary bladder: a clinicopathological study of 11 cases. J Clin Pathol. 2000;53:458–61. 10.1136/jcp.53.6.458. 10911804 10.1136/jcp.53.6.458 PMC1731210

[7] Simpson WG, Lopez A, Babbar P, Payne LF. Primary bladder lymphoma, diffuse large B-cell type: case report and literature review of 26 cases. Urol Ann. 2015 Apr-Jun;7(2):268–72. 10.4103/0974-7796.152947. 10.4103/0974-7796.152947 PMC4374275 25837971

[8] Chuang T-Y, Chang T-W, Chen S-S, Chang C-C, Cheng W-M, Wei Y-H. Mitochondrial dysfunction in patients with urogenital disease. Urol Sci 2021 Oct–Dec 32(4):p 143–50; 10.4103/UROS.UROS_47_21

[9] Isaacson PG, Du MQ. MALT lymphoma: from morphology to molecules. Nat Rev Cancer. 2004;4:644–53. 10.1038/nrc1409. 15286744 10.1038/nrc1409

[10] Vempati P, Knoll MA, Alqatari M, Strauchen J, Malone AK, Bakst RL. MALT lymphoma of the bladder: a Case Report and Review of the literature. Case Rep Hematol. 2015;2015:9343. 10.1155/2015/934374. 10.1155/2015/934374 PMC4568333 26417464

